# Supplementary material for: Single-cell transcriptomics defines an improved, validated monoculture protocol for differentiation of human iPSC to microglia
Source: Sci Rep. 2022 Nov 14;12:19454. doi: 10.1038/s41598-022-23477-2 (PMC9663826; doi:10.1038/s41598-022-23477-2)
Supplement: Supplementary file 1 — Supplementary Figures. [file 41598_2022_23477_MOESM1_ESM.pdf]

SUPPLEMENTARY FIGURES

Supplementary figure 1: Development of the pmChGIP SH-SY5Y cell line for measuring autophagy. **A)** The pmChGIP construct was generated by PCR amplification of mCherry-eGFP fusion from the pBABE-puro-mCherry-eGFP-LC3B vector with SpeI and BamHI restriction sites. These were cloned into a lentiviral backbone EF1α IRES Puro to generate the pmChGIP vector. **B)** Plasmid map of pmChGIP, **C)** HEK293T were transfected with pmChGIP (dual mCherry eGFP vector, pmCherry (single mCherry vector), or pGIP (single eGFP vector) to confirm dual expression. Images taken at 48hr post transfection using EVOS Floid at 20x magnification. Scale bar 100µm. Cont are untransfected HEK293T cells. **D)** SH-SY5Y at 48hr following transduction with pmChGIP lentivirus confirms successful integration and signal. Images taken using EVOS Floid at 20x magnification. Scale bar 100µm. Cont are untransduced SH-SY5Y. **E)** Clonal pmChGIP SH-SY5Y cell lines were generated by transduction of pmChGIP at 1:32 or 1:2 dilution of lentivirus (corresponding to MOI of 0.3 and 2 respectively) into p10 SH-SY5Y. After 24hr single cell sorting was undertaken for double positive cells. Sorted cells were expanded over several weeks before banking. Fluorescence of the four 1:32 pmChGIP SH-SY5Y lines and two 1:2 pmChGIP SH-SY5Y lines were quantified using FACS. Cells were either live or fixed, to mimic the phagocytosis assay conditions. Fixing reduced the fluorescent intensity of all lines as expected. Line pmChGIP 1:2 F4 showed the highest geometric mean intensities and was used for phagocytosis assays. **F)** Example FACS plots showing clear separation of control SH-SY5Y and the pmChGIP 1:2 F4 SH-SY5Y cell line. 488 signal on the X-axis and 561 signal on the Y-axis.

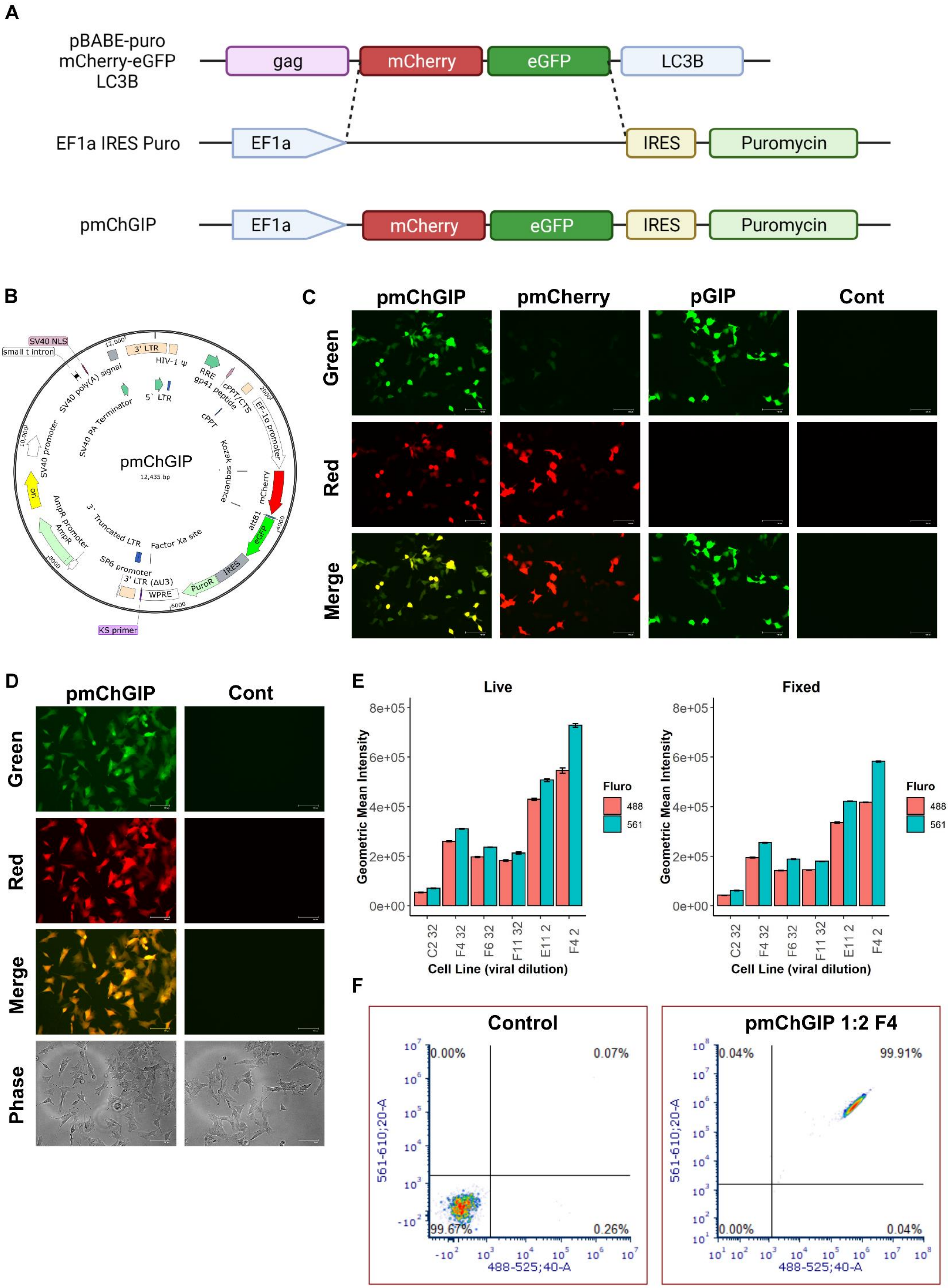
A
pBABE-puro mCherry-eGFP LC3B
gag
mCherry
eGFP
LC3B
EF1a IRES Puro
EF1a
IRES
Puromycin
pmChGIP
B
pmChGIP
12,435 bp
C
pmChGIP
pmCherry
pGIP
Cont
Green
Red
Merge
D
Phase
E
Live
Fixed
Geometric Mean Intensity
Fluro
488
561
Cell Line (viral dilution)
C2 32
F4 32
F6 32
F11 32
E11 2
F4 2
F
Control
pmChGIP 1:2 F4
561-610;20-A
488-525;40-A
0.00%
0.07%
99.67%
0.26%
0.04%
99.91%
0.00%
0.04%

Supplementary figure 2: Overlapping qPCR profiles of IMBN and ITGBN from two different institutes with different genetic backgrounds confirms reproducibility. ddCT values for iPSC-microglia cultured in IMBN **A)** and ITGBN **B)** at both Oxford and Cambridge using different genetic backgrounds show a strong correlation in their expression profiles. ddCT values calculated against IGBN. (IMBN, R^2=0.236, p-value=0.03. ITGBN, R^2=0.348, p-value=0.006).

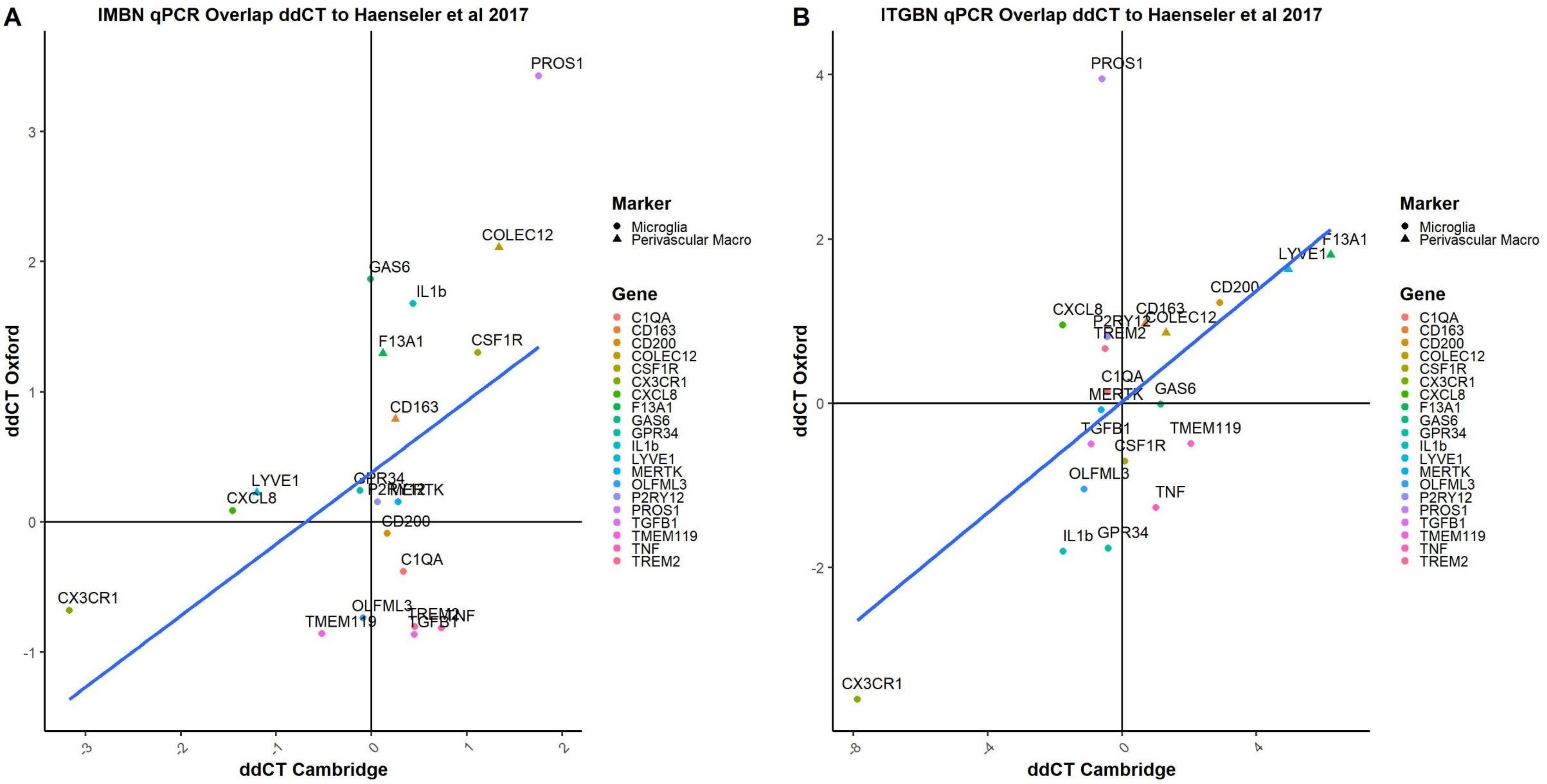
A
IMBN qPCR Overlap ddCT to Haenseler et al 2017
ddCT Oxford
ddCT Cambridge
PROS1
COLEC12
GAS6
IL1b
F13A1
CSF1R
CD163
LYVE1
GPR34
CXCL8
CD200
C1QA
CX3CR1
OLFML3
TMEM119
TGFB1
B
ITGBN qPCR Overlap ddCT to Haenseler et al 2017
ddCT Oxford
ddCT Cambridge
PROS1
F13A1
LYVE1
CD200
CXCL8
CD163
P2RY12
COLEC12
TREM2
C1QA
MERTK
GAS6
TGFB1
TMEM119
CSF1R
OLFML3
TNF
IL1b
GPR34
CX3CR1
Marker
Microglia
Perivascular Macro
Gene
C1QA
CD163
CD200
COLEC12
CSF1R
CX3CR1
CXCL8
F13A1
GAS6
GPR34
IL1b
LYVE1
MERTK
OLFML3
P2RY12
PROS1
TGFB1
TMEM119
TNF
TREM2

Supplementary figure 3: Overlapping qPCR profiles of ITMG and ITM from two different institutes with different genetic backgrounds confirms reproducibility. ddCT values for iPSC-microglia cultured in ITMG **A)** and ITM ADMEM **B)** at both Oxford and Cambridge using different genetic backgrounds show a strong correlation in their expression profiles. ddCT values calculated against microglial precursors. (ITM, R^2=0.639, p-value=$2.36x10^{-5}$, ITMG, R^2 =0.422, p-value=0.00195).

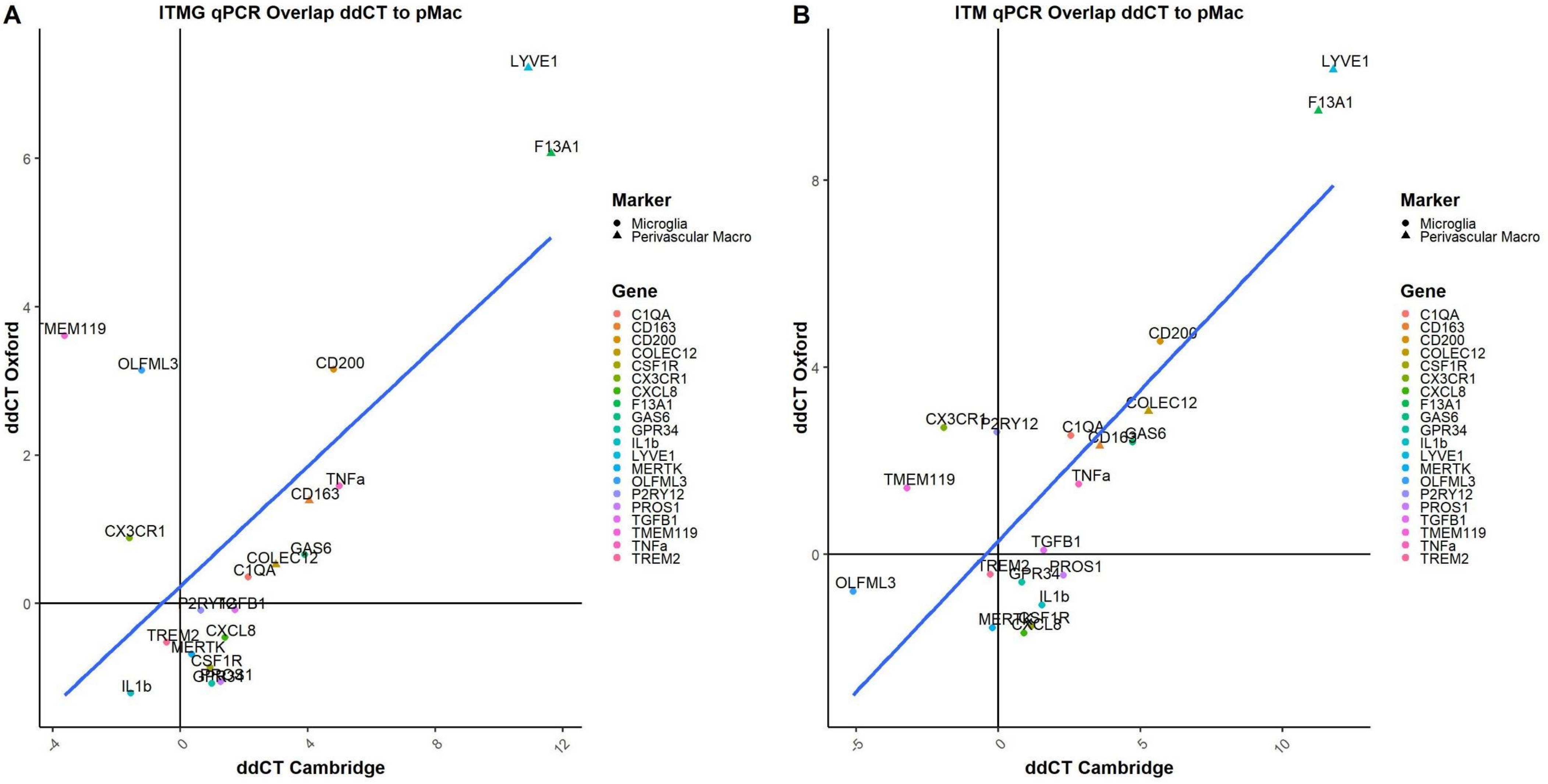
A
ITMG qPCR Overlap ddCT to pMac
ddCT Oxford
ddCT Cambridge
LYVE1
F13A1
TMEM119
OLFML3
CD200
TNFa
CD163
CX3CR1
GAS6
COLEC12
C1QA
P2RY12
TGFB1
CXCL8
TREM2
MERTK
CSF1R
PROS1
GPR34
IL1b
Marker
Microglia
Perivascular Macro
Gene
C1QA
CD163
CD200
COLEC12
CSF1R
CX3CR1
CXCL8
F13A1
GAS6
GPR34
IL1b
LYVE1
MERTK
OLFML3
P2RY12
PROS1
TGFB1
TMEM119
TNFa
TREM2
B
ITM qPCR Overlap ddCT to pMac
ddCT Oxford
ddCT Cambridge
LYVE1
F13A1
CD200
COLEC12
CX3CR1
P2RY12
C1QA
CD163
GAS6
TMEM119
TNFa
TGFB1
TREM2
PROS1
GPR34
OLFML3
IL1b
MERTK
CSF1R
CXCL8
Marker
Microglia
Perivascular Macro
Gene
C1QA
CD163
CD200
COLEC12
CSF1R
CX3CR1
CXCL8
F13A1
GAS6
GPR34
IL1b
LYVE1
MERTK
OLFML3
P2RY12
PROS1
TGFB1
TMEM119
TNFa
TREM2

Supplementary figure 4: Representative images of iPSC-microglia differentiated for 14d on three different genetic backgrounds (SFC841-03-01, SFC856-03-04, KOLF2.1S). Images taken at d14 at 10x magnification. Scale bar 100µm.

KOLF2.1S SFC-841-03-01 SFC856-03-04

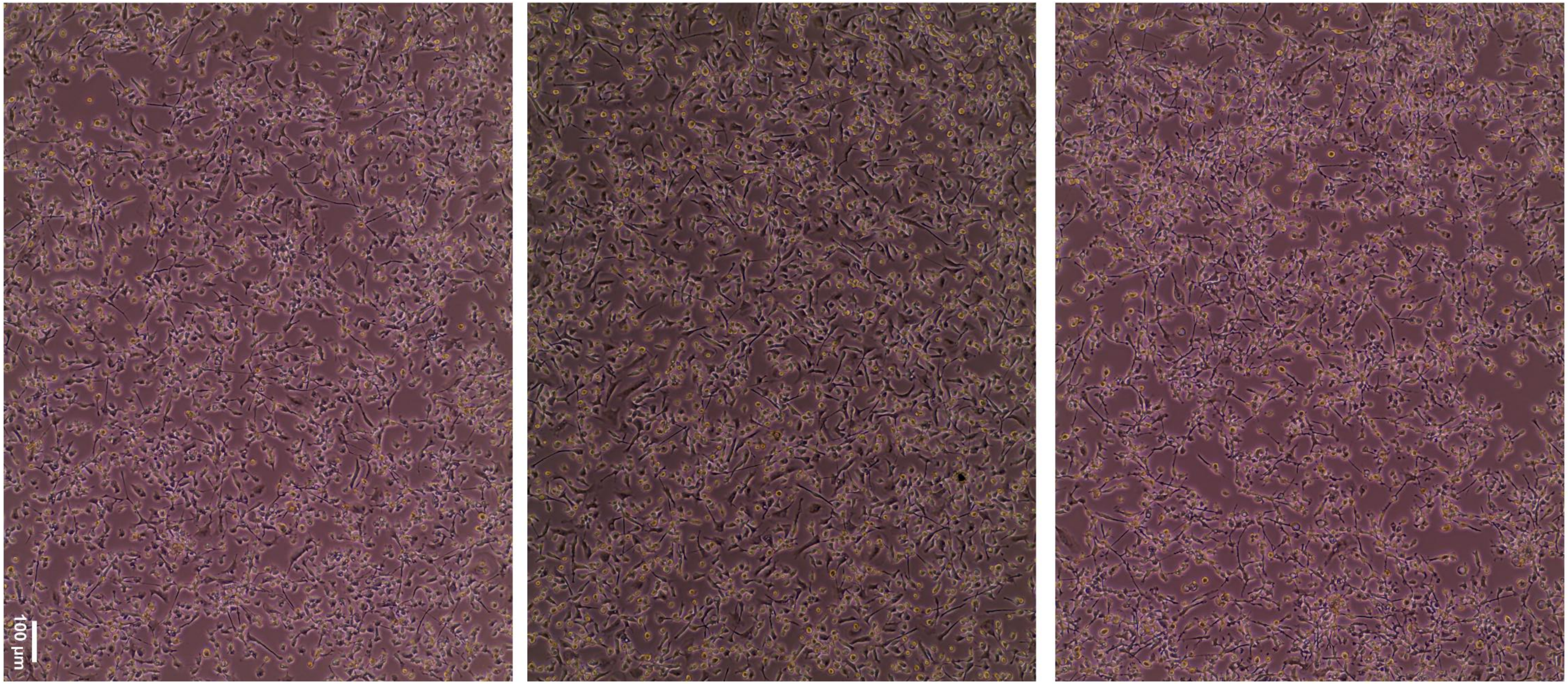

Supplementary figure 5: Comparison of single cell RNA-seq analysis of six media to external data. A) Principal component analysis (PCA) visualization of the six media samples from this manuscript (“Ours” after pseudobulk aggregation) compared to external bulk RNA-seq data (“Abud”, ”Douvaras”, ”Galatro”, “Mancuso”, ”Muffat”), represented with different shapes. Cell types are highlighted with different colours: “exBrain”: ex vivo brain cortex tissue; “exDC”, ex vivo dendritic cells; “exMAC”, ex vivo macrophages; “exMGL”, ex vivo microglia; “exMonocyte”, ex vivo monocyte; “iMG”, iPSC-derived microglia; “iMG_neuron”, iPSC-derived microglia co-cultured with neurons; “iNPC”, iPSC-derived neuronal progenitors; “iPSC”, induced pluripotent stem cells; “pAMGL”, primary adult microglia; “pAMGL”, pAMGL + serum; “pFMGL”, primary foetal microglia; “pMAC”, primary macrophages. A few sample points have been labelled directly for clarity. **B)** A highlighted version with only our samples and primary microglia colored (in red and black respectively).

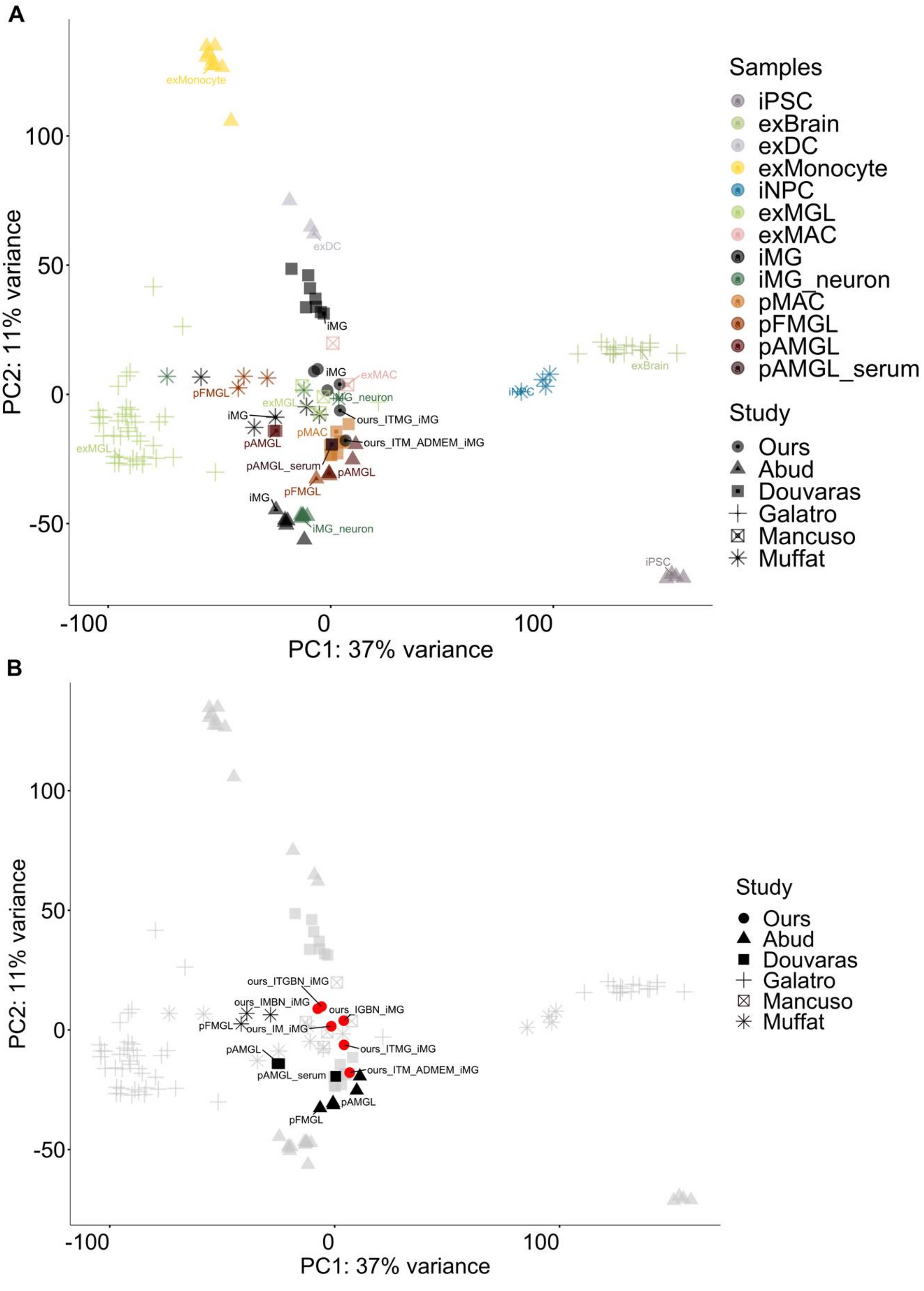
A
Samples
iPSC
exBrain
exDC
exMonocyte
iNPC
exMGL
exMAC
iMG
iMG_neuron
pMAC
pFMGL
pAMGL
pAMGL_serum
Study
Ours
Abud
Douvaras
Galatro
Mancuso
Muffat
exMonocyte
exDC
iMG
exMAC
exBrain
iNPC
pFMGL
exMGL
iMG_neuron
ours_ITMG_iMG
pAMGL
pMAC
ours_ITM_ADMEM_iMG
pAMGL_serum
pAMGL
pFMGL
iMG_neuron
iPSC
PC1: 37% variance
PC2: 11% variance
B
Study
Ours
Abud
Douvaras
Galatro
Mancuso
Muffat
ours_ITGBN_iMG
ours_IMBN_iMG
ours_IGBN_iMG
pFMGL
ours_IM_iMG
ours_ITMG_iMG
pAMGL
pAMGL_serum
ours_ITM_ADMEM_iMG
pAMGL
pFMGL
PC1: 37% variance
PC2: 11% variance

Supplementary figure 6: Single cell RNA-seq analysis of six media. A) UMAP visualizations of cells coloured by clusters or inferred cell cycle phase (top), and principal component analysis (PCA) visualizations of cells coloured and split by media (bottom). **B)** UMAP visualizations of cells coloured by gene expression density of perivascular macrophage and microglial marker genes. **D)** Intersecting set plot of differentially expressed genes between ITGBN, IMBN, IM, ITMG and ITM ADMEM media against the IGBN baseline. Total differentially expressed genes per contrast in “Set Size”. **E)** UMAP visualizations of cells coloured by label transfer assignments per training data.

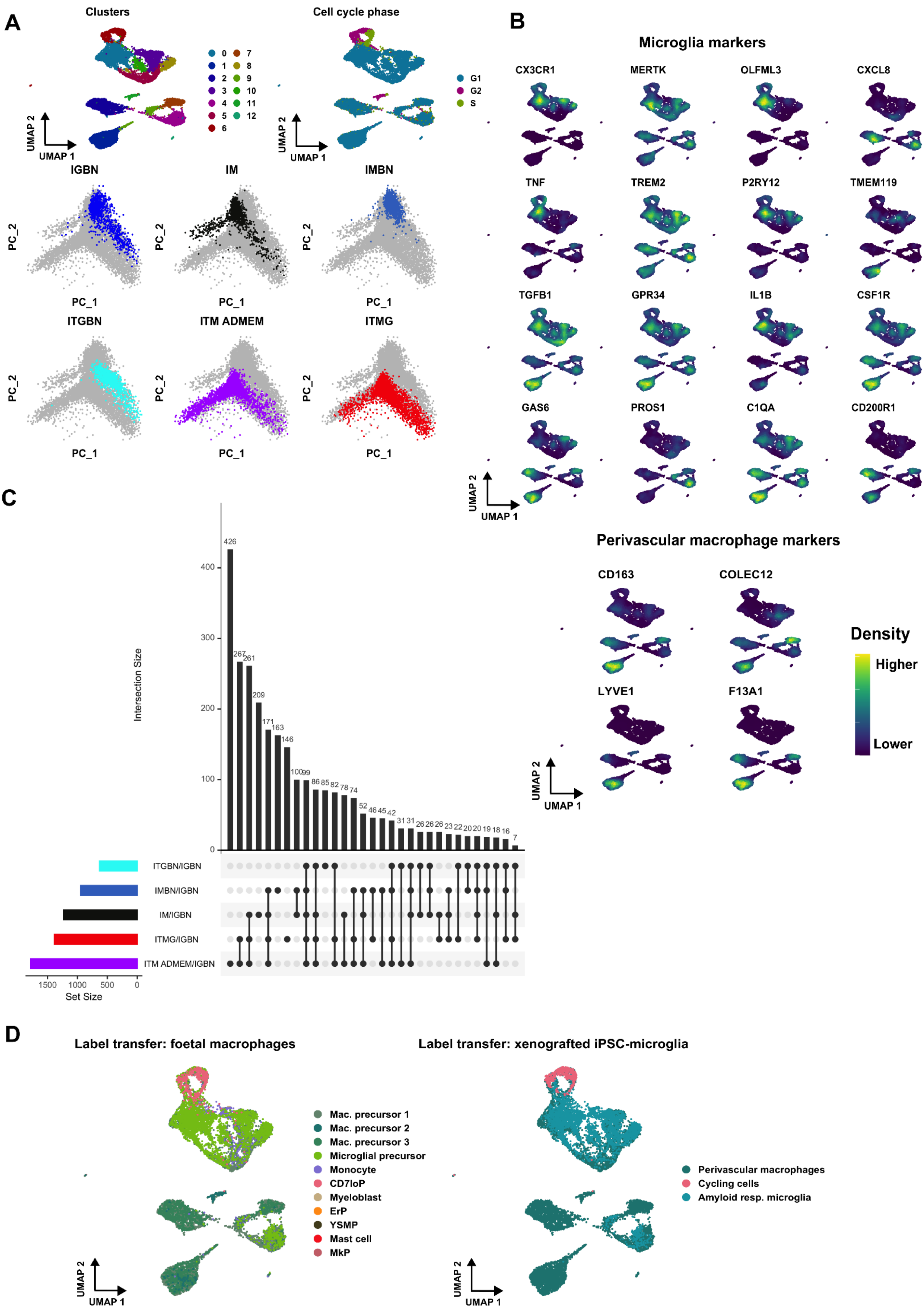
A
Clusters
Cell cycle phase
G1
G2
S
UMAP 1
UMAP 2
IGBN
IM
IMBN
ITGBN
ITM ADMEM
ITMG
PC_1
PC_2
B
Microglia markers
CX3CR1
MERTK
OLFML3
CXCL8
TNF
TREM2
P2RY12
TMEM119
TGFB1
GPR34
IL1B
CSF1R
GAS6
PROS1
C1QA
CD200R1
Perivascular macrophage markers
CD163
COLEC12
LYVE1
F13A1
Density
Higher
Lower
C
Intersection Size
Set Size
ITGBN/IGBN
IMBN/IGBN
IM/IGBN
ITMG/IGBN
ITM ADMEM/IGBN
D
Label transfer: foetal macrophages
Label transfer: xenografted iPSC-microglia
Mac. precursor 1
Mac. precursor 2
Mac. precursor 3
Microglial precursor
Monocyte
CD7loP
Myeloblast
ErP
YSMP
Mast cell
MkP
Perivascular macrophages
Cycling cells
Amyloid resp. microglia
